# Triple Colonic Fistula Due to Complicated Diverticular Disease and Its Surgical Management

**DOI:** 10.7759/cureus.76623

**Published:** 2024-12-30

**Authors:** Noe Perez-Carrillo, Ricardo O'Farril-Anzures, Naomi Contreras Galván, Adelina Rojas-Granados, Manuel Angeles-Castellanos

**Affiliations:** 1 Department of Neurosurgery, Hospital General de Mexico Dr. Eduardo Liceaga, Mexico City, MEX; 2 Department of Coloproctology, Hospital de Alta Especialidad Centro Sur de Petróleos Mexicanos, Mexico City, MEX; 3 Faculty of Medicine, Universidad Nacional Autonoma de Mexico, Mexico City, MEX; 4 Department of Anatomy, Faculty of Medicine, Universidad Nacional Autonoma de Mexico, Mexico City, MEX

**Keywords:** colon, diverticular disease, fistula, pneumaturia, rectosigmoid junction

## Abstract

Diverticular colon disease is the most common cause of colovesical fistulas, a rare and complex entity in their diagnosis and treatment. This report details the case of a 56-year-old patient who had presented with pneumaturia and gas in the vagina for six years and exudate in the abdominal wall in a midline wound. Given the suspicion, the diagnosis of the triple colonic fistula was confirmed by imaging studies: enteroatmospheric, colovesical, and colotubal, which were managed surgically.

## Introduction

Diverticular disease is a common condition in Western countries, and its incidence increases with age (occurring in about 5% of people under 40 years of age and 50% around 60 years of age) and increases to more than 71% in people over 80 years of age, with a similar prevalence in both men and women [1,2].

Diverticular disease is classified as asymptomatic and symptomatic; within the latter, there is the subclassification of uncomplicated and complicated. Similarly, diverticulitis is classified as complicated and uncomplicated [3]. Complications include perforations, abscesses, fistulas, and mechanical ileus [4,5].

The rate of new-onset fistulas after acute diverticulitis with abscesses is around 14% [6]. Sigmoid diverticulitis can lead to the formation of fistulas towards the urinary bladder, ureters, uterus, vagina, and skin, especially in the perianal region, as well as cholenteric and coloenteric fistulas, but the presence of two or more fistulas are relatively uncommon conditions, most frequently resulting from diverticular disease, and this explains the relevance of the case we present since the medical and surgical management is much more complicated. Cholovesical fistulas are the most frequent [4].

We present the case of a 56-year-old female patient with a triple colonic fistula due to complicated diverticular disease.

## Case presentation

We present the case of a 56-year-old female patient, with a history of smoking, no chronic degenerative diseases, and previously unknown colonic diverticular disease. She had a right salpingo-oophorectomy 37 years ago due to an ectopic pregnancy, a midline approach cesarean section 30 years ago, conventional cholecystectomy 29 years ago, and a hysterectomy 15 years ago due to uterine fibroids. One year ago, she underwent an exploratory laparotomy and surgical drainage for an abdominal wall abscess.

**Figure 1:**
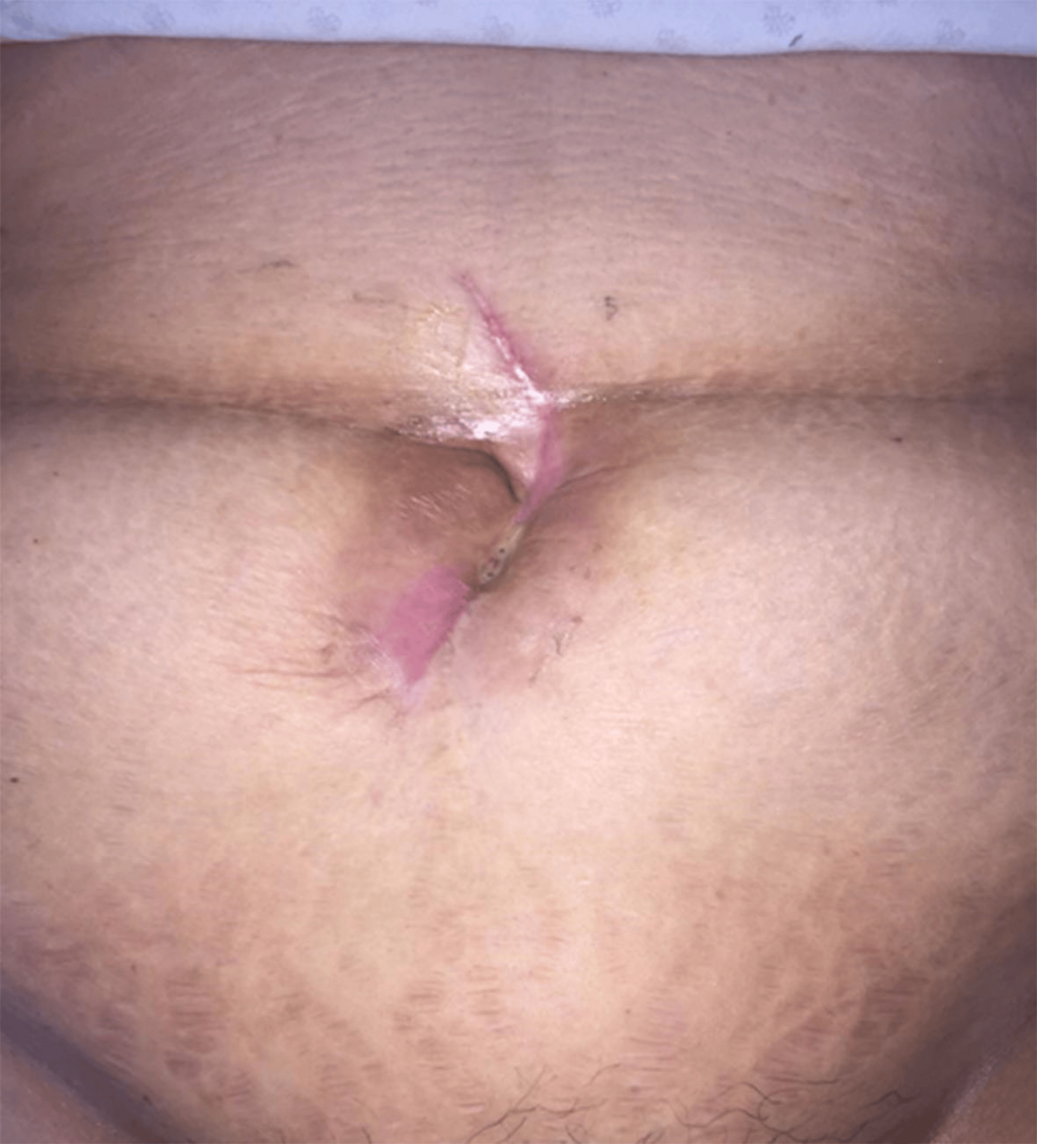
Abdominal scar with evident yellowish exudate over the fistulous opening.

The patient was evaluated in our clinic for pneumaturia and the presence of gas discharge from the vagina with a six-year evolution. She also reported pain at the abdominal scar, which subsided with the drainage of fetid exudate. Physical examination revealed a visible 5-mm fistulous opening at the infraumbilical region with granulation tissue and scant yellowish exudate, without abdominal pain on superficial or deep palpation (Figure 1).

**Figure 2:**
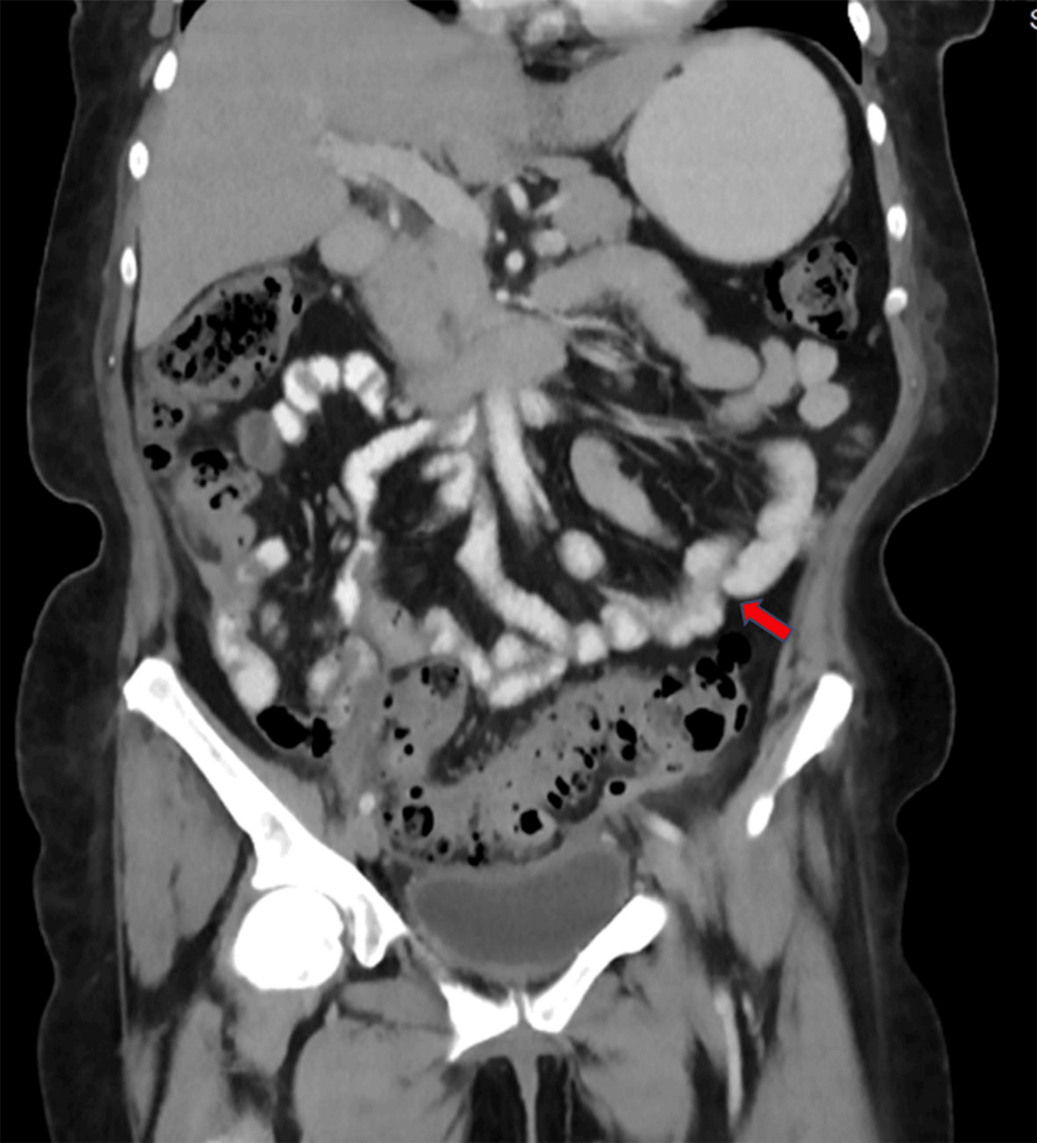
Small diverticular sacs and a large one with a high risk of imminent perforation (arrow).

**Figure 3:**
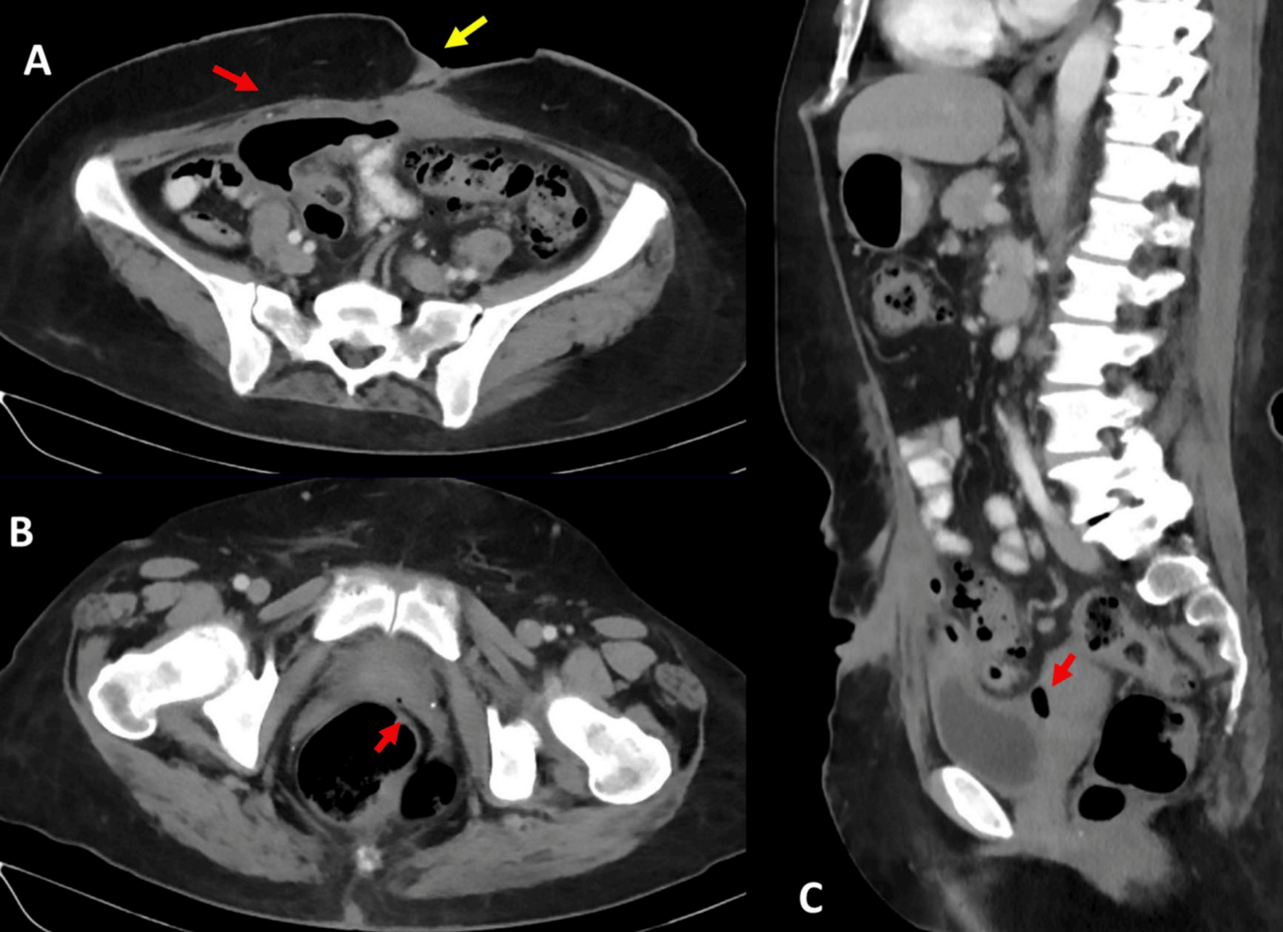
A) Entero-atmospheric fistula, gas in the intraperitoneal space (red arrow), and fistulous tract opening in the midline of the abdominal wall (yellow arrow); B) colo-vesical fistula, intravesical gas with a fistulous tract originating from the intestinal lumen (arrow); C) suggestive image of colo-tubal fistula evidenced by the presence of gas in the pouch of Douglas (arrow).

Given the patient's history and clinical presentation suggesting a triple colonic fistula, an evaluation was initiated with double-contrast computed tomography (CT) scans. The etiology of diverticular disease was identified (Figure 2), along with a significant amount of air in the intraperitoneal space with a fistulous tract towards the defect in the midline abdominal wall, making the entero-atmospheric fistula evident (Figure 3A).

Additionally, intravesical air with a tract to the colon was observed (Figure 3B), as well as air in the pouch of Douglas (Figure 3C).

Once the diagnosis was established, preoperative surgical planning was carried out, assessing aspects according to the American College of Surgeons STRONG guidelines, focusing on four areas: nutrition, smoking, glycemia, and medication management. The patient was admitted the day before surgery for bowel preparation with an osmotic laxative, mechanical prophylaxis according to thromboembolism risk using the Caprini scale, and an eight-hour fasting period following the institutional anesthesiologist protocol. For antibiotic prophylaxis, we used cephalosporin and metronidazole according to the Stanford antimicrobial guidelines.

Procedure

**Figure 4:**
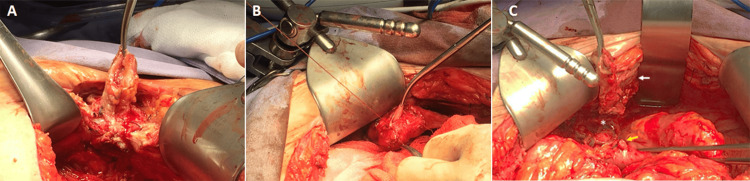
A) Dissection of colocutaneous fistula; B) fistulous tract to the bladder; C) entero-atmospheric fistula (white arrow). Closure of colo-vesical fistula opening (yellow arrow), colo-tubal fistula (asterisk).

A midline incision was made, and the dissection was carried out layer by layer from the surface to the depth until reaching the abdominal cavity. Extensive adhesiolysis was performed, and the entero-atmospheric fistula tract was resected (Figure 4A). The fistulous tract from the colon to the bladder was then dismantled, and the bladder wall was repaired with simple interrupted chromic sutures (Figure 4B). Finally, the entire sigmoid tract from the skin to the fallopian tube, where the third colo-tubal fistula was located, was resected (Figure 4C). A sigmoidectomy was then performed, with resection of the rectosigmoid junction using a Contour® curved cutter stapler and colorectal anastomosis with a 31-mm circular stapler.

The postoperative evolution was satisfactory, with no evidence of fistulous tracts or gas leakage through the vagina and bladder, allowing the patient to return to her daily activities.

## Discussion

The incidence of diverticular disease, although considered a rare complication, has increased in recent years, and the overall incidence of complex diverticulitis has increased, leading to an increase in symptomatic disease [1,7].

The diagnosis of fistulas due to diverticular disease is complex and remains a challenge. Recurrent urinary tract infections, pollakiuria, dysuria, and hematuria are the most frequent clinical findings. Although the patient in our case presented pneumaturia and fecaluria, these two symptoms have been considered pathognomonic of fistulas; however, these symptoms are not always present, as demonstrated in a series of 49 patients, where only 71.4% of the patients presented pneumaturia and 51% fecaluria [8-10].

On the other hand, some authors have considered the need for treatment of chronic infection, the threat of urosepsis, as well as the decreased risk of eventual renal loss [9,10]. Once surgical treatment is decided, standard treatment focuses on identifying and dividing the fistula, resecting the affected portion of the colon, and repairing all involved structures if necessary [11].

While excision of the affected colon (most commonly the sigmoid) has been shown to be necessary due to the high recurrence rate with a simple division of the fistula without resection, in our patient, an exploratory laparotomy was performed, observing a plastron in the pelvic cavity with great adherence of the sigmoid to the fistula site and posterior bladder wall; adhesiolysis and identification of the fistulous tract with sharp dissection were performed, the fistula was detached from the entire abdominal wall towards the colon, as well as left salpingectomy, as mentioned in the results, and currently the patient is asymptomatic.

While most surgeons agree that the presence of a colovesical fistula is an indication for surgery, some clinical situations require reconsideration, particularly in the setting of an asymptomatic patient with multiple comorbidities, and medical treatment may be the best option [12].

## Conclusions

There are various treatments for enteric fistulas that do not resolve spontaneously. The importance of a multidisciplinary approach to planning a successful surgery with complete lesion resection should always consider, as much as possible, eliminating the infection and malnutrition factors. This case report intends to share our experience obtained during the two-year follow-up of this patient in hopes that more experiences will be gathered worldwide to standardize the therapeutic approach to be followed.
